# Supplementary figures and images for: Assessment of the clinical utility of four NGS panels in myeloid malignancies. Suggestions for NGS panel choice or design
Source: PLoS One. 2020 Jan 24;15(1):e0227986. doi: 10.1371/journal.pone.0227986 (PMC6980571; doi:10.1371/journal.pone.0227986)

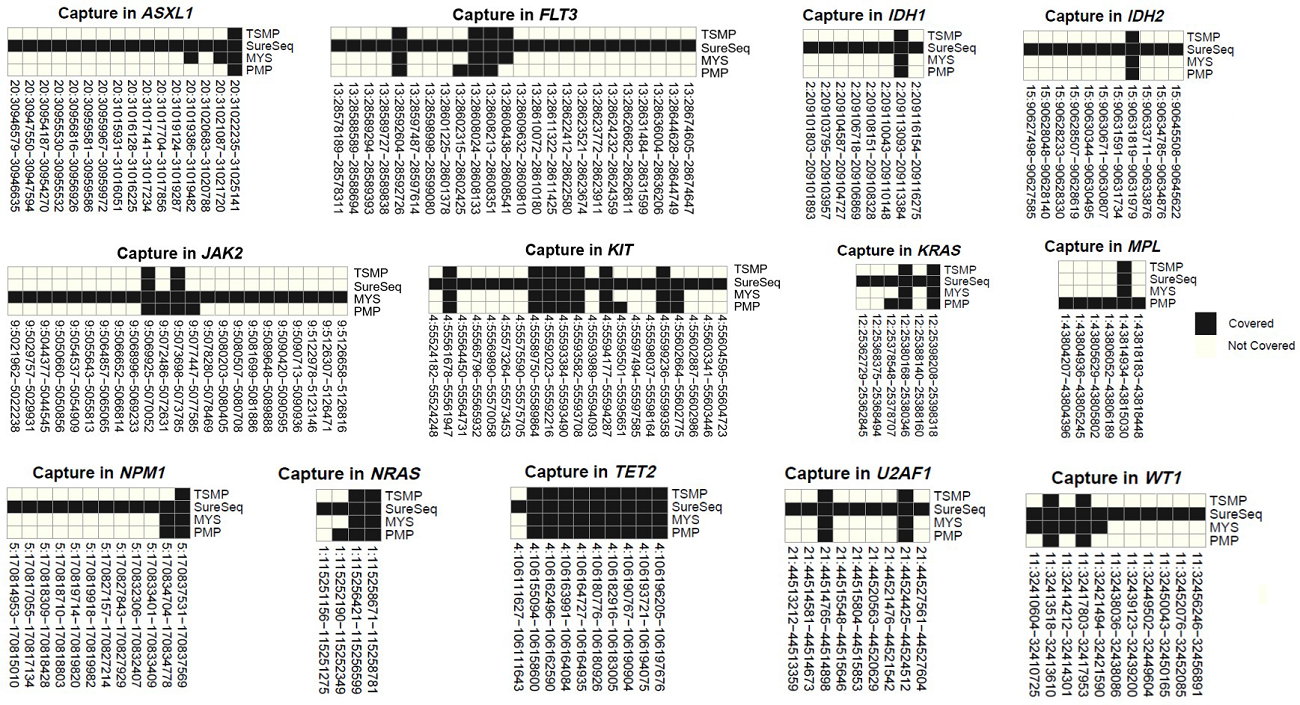

Supplement: S1 Fig — SureSeq panels design included a larger target region of ASXL1, FLT3, IDH1, IDH2, KIT, KRAS, NPM1, NRAS, TET2, U2AF1 and WT1 genes, whereas JAK2 gene was more widely covered by MYS panel, and MPL gene by PMP. (TIF) [file pone.0227986.s001.tif]

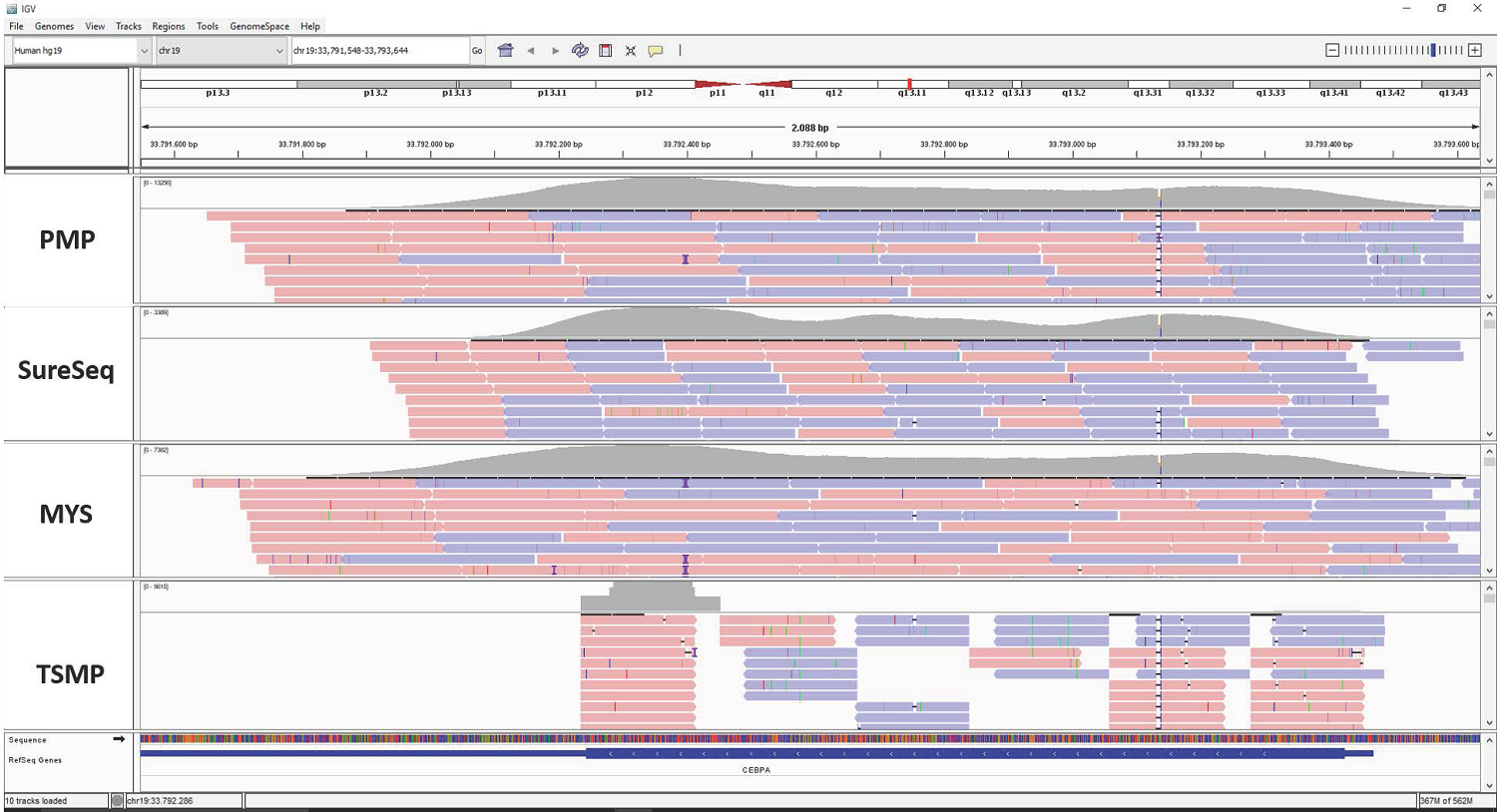

Supplement: S3 Fig — IGV screenshot showing genomic position (top track), CEBPA gene structure (bottom track) and coverage for the different panels (four central tracks). Panel tracks show differential coverage in grey color, and reads 1 and 2 in red and blue bars. TSMP track shows poor and heterogeneous coverage for CEBPA gene. (TIF) [file pone.0227986.s003.tif]

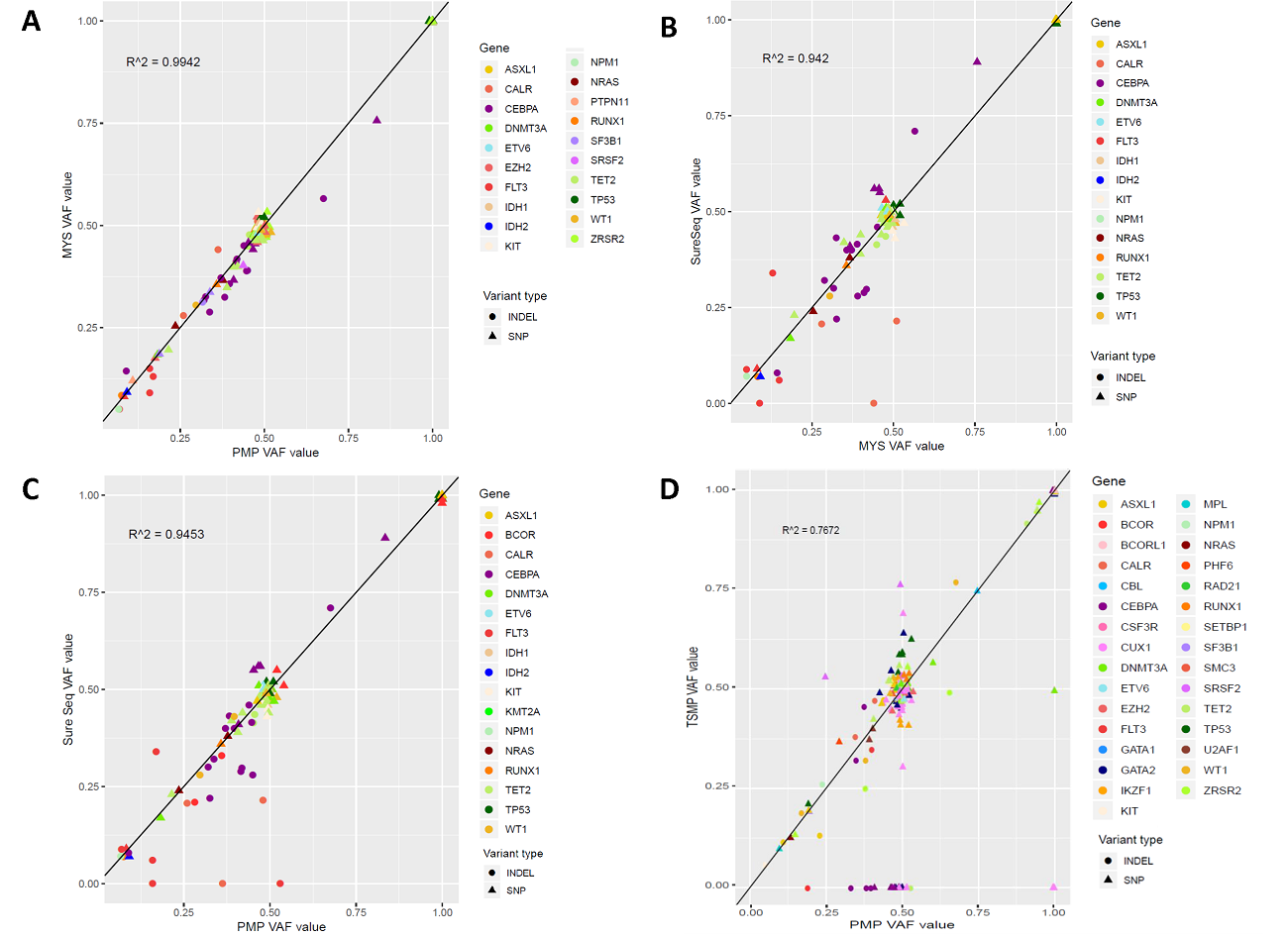

Supplement: S5 Fig — A: Comparison between variants called by PMP and MYS panel in their 27 genes in common. B: Comparison between variants called by PMP and SureSeq panels in their 23 genes in common. C: Comparison between variants called by MYS and SureSeq panels in their 19 genes in common. D: Comparison between variants called by PMP and TSMP in their 40 genes in common. (TIFF) [file pone.0227986.s005.tiff]
