# Supplementary material for: Assessment of the clinical utility of four NGS panels in myeloid malignancies. Suggestions for NGS panel choice or design
Source: PLoS One. 2020 Jan 24;15(1):e0227986. doi: 10.1371/journal.pone.0227986 (PMC6980571; doi:10.1371/journal.pone.0227986)
Supplement: S1 Table — TSMP includes a total of 54 genes for SNV and indels. (DOCX) [file pone.0227986.s006.docx]

**S1 Table. TruSight Myeloid Panel (TSMP) target regions per gene.** TSMP includes a total of 54 genes for SNV and indels.

| **GENE** | **Transcript** | **ENSEMBL** | **EXONS Target Region** |
| --- | --- | --- | --- |
| ***ABL1*** | NM_007313.2 | ENST00000318560 | 4, 5, 6 |
| ***ASXL1*** | NM_015338.5 | ENST00000375687 | 12 |
| ***ATRX*** | NM_000489.3 | ENST00000373344 | 8, 10, 17‐31 |
| ***BCOR*** | NM_001123385.1 | ENST00000397354 | 2‐15 |
| ***BCORL1*** | NM_021946.4 | ENST00000540052 | 1‐12 |
| ***BRAF*** | NM_004333.4 | ENST00000288602 | 15 |
| ***CALR*** | NM_004343.3 | ENST00000316448 | 9 |
| ***CBL*** | NM_005188.3 | ENST00000264033 | 8, 9 |
| ***CBLB*** | NM_170662.3 | ENST00000264122 | 9, 10 |
| ***CBLC*** | NM_012116.3 | ENST00000270279 | 9 |
| ***CDKN2A*** | NM_001195132.1 | ENST00000304494 | 1‐3 |
| ***CEBPA*** | NM_004364.3 | ENST00000498907 | 1 |
| ***CSF3R*** | NM_156039.3 | ENST00000373103 | 14‐17 |
| ***CUX1*** | NM_001202543.1 | ENST00000360264 | 1‐24 |
| ***DNMT3A*** | NM_022552.4 | ENST00000264709 | 1‐23 |
| ***ETV6*** | NM_001987.4 | ENST00000396373 | 1‐8 |
| ***EZH2*** | NM_004456.4 | ENST00000320356 | 2‐20 |
| ***FBXW7*** | NM_033632.3 | ENST00000281708 | 9‐11 |
| ***FLT3*** | NM_004119.2 | ENST00000241453 | 13 (parcial), 14, 15, 20 |
| ***GATA1*** | NM_002049.3 | ENST00000376670 | 2 |
| ***GATA2*** | NM_032638.4 | ENST00000341105 | 2‐6 |
| ***GNAS*** | NM_080425.2 | ENST00000371085 | 8, 9, 10 (parcial) |
| ***HRAS*** | NM_005343.2 | ENST00000451590 | 2, 3 |
| ***IDH1*** | NM_005896.2 | ENST00000415913 | 4 |
| ***IDH2*** | NM_002168.2 | ENST00000330062 | 4 |
| ***IKZF1*** | NM_001220765.1 | ENST00000331340 | 2‐8 |
| ***JAK2*** | NM_004972.3 | ENST00000381652 | 12, 14 |
| ***JAK3*** | NM_000215.3 | ENST00000458235 | 13 |
| ***KDM6A*** | NM_021140.2 | ENST00000377967 | 1‐29 |
| ***KIT*** | NM_000222.2 | ENST00000288135 | 2, 8‐11, 13, 17 |
| ***KRAS*** | NM_033360.2 | ENST00000256078 | 2, 3 |
| ***KMT2A*** | NM_001197104.1 | ENST00000534358 | 5‐7 (parcial), 8 |
| ***MPL*** | NM_005373.2 | ENST00000372470 | 10 |
| ***MYD88*** | NM_001172566 | ENST00000396334 | 3, 4, 5 |
| ***NOTCH1*** | NM_017617.3 | ENST00000277541 | 26, 27, 28, 34 |
| ***NPM1*** | NM_002520.6 | ENST00000296930 | 11 |
| ***NRAS*** | NM_002524.4 | ENST00000369535 | 2, 3 |
| ***PDGFRA*** | NM_006206.4 | ENST00000257290 | 12, 14, 18 |
| ***PHF6*** | NM_032458.2 | ENST00000332070 | 2‐10 |
| ***PTEN*** | NM_000314.4 | ENST00000371953 | 5, 7 |
| ***PTPN11*** | NM_002834.3 | ENST00000351677 | 3, 13 |
| ***RAD21*** | NM_006265.2 | ENST00000297338 | 2‐14 |
| ***RUNX1*** | NM_001754.4 | ENST00000437180 | 1‐9 |
| ***SETBP1*** | NM_001130110.1 | ENST00000282030 | 4 (parcial) |
| ***SF3B1*** | NM_012433.2 | ENST00000335508 | 13‐16 |
| ***SMC1A*** | NM_006306.2 | ENST00000322213 | 2, 11, 16, 17 |
| ***SMC3*** | NM_005445.3 | ENST00000361804 | 10, 13 (parcial), 19, 23, 25, 28 |
| ***SRSF2*** | NM_003016 | ENST00000392485 | 1 |
| ***STAG2*** | NM_001042749.1 | ENST00000218089 | 3‐35 |
| ***TET2*** | NM_001127208.2 | ENST00000380013 | 3‐11 |
| ***TP53*** | NM_000546.5 | ENST00000269305 | 2‐11 |
| ***U2AF1*** | NM_001025203.1 | ENST00000291552 | 2, 6 |
| ***WT1*** | NM_024426.4 | ENST00000332351 | 7, 9 |
| ***ZRSR2*** | NM_005089.3 | ENST00000307771 | 1‐11 |
