# Supplementary material for: Assessment of the clinical utility of four NGS panels in myeloid malignancies. Suggestions for NGS panel choice or design
Source: PLoS One. 2020 Jan 24;15(1):e0227986. doi: 10.1371/journal.pone.0227986 (PMC6980571; doi:10.1371/journal.pone.0227986)
Supplement: S2 Table — MYS panel design includes a total of 30 genes for SNV and indels. (DOCX) [file pone.0227986.s007.docx]

**S2 Table. Myeloid Solutions Panel (MYS) target regions per gene.** MYS panel design includes a total of 30 genes for SNV and indels.

| **GENE** | **Transcript** | **ENSEMBL** | **EXONS Target Region** |
| --- | --- | --- | --- |
| ***ABL1*** | NM_005157 | ENST00000318560 | 4-9 |
| ***ASXL1*** | NM_015338 | ENST00000375687 | 9,11,12 |
| ***BRAF*** | NM_004333 | ENST00000288602 | 15 |
| ***CALR*** | NM_004343 | ENST00000316448 | 9 |
| ***CBL*** | NM_005188 | ENST00000264033 | 8,9 |
| ***CEBPA*** | NM_004364 | ENST00000498907 | all |
| ***CSF3R*** | NM_000760 | ENST00000373103 | all |
| ***DNMT3A*** | NM_022552 | ENST00000264709 | all |
| ***ETV6*** | NM_001987 | ENST00000396373 | all |
| ***EZH2*** | NM_004456 | ENST00000320356 | all |
| ***FLT3*** | NM_004119 | ENST00000241453 | 13-15,20 |
| ***HRAS*** | NM_005343 | ENST00000451590 | 2,3 |
| ***IDH1*** | NM_005896 | ENST00000415913 | 4 |
| ***IDH2*** | NM_002168 | ENST00000330062 | 4 |
| ***JAK2*** | NM_004972 | ENST00000381652 | all |
| ***KIT*** | NM_000222 | ENST00000288135 | 2,8-11,13,17,18 |
| ***KRAS*** | NM_004985 | ENST00000256078 | 2,3 |
| ***MPL*** | NM_005373 | ENST00000372470 | 10 |
| ***NPM1*** | NM_002520 | ENST00000296930 | 10,11 |
| ***NRAS*** | NM_002524 | ENST00000369535 | 2,3 |
| ***PTPN11*** | NM_002834 | ENST00000351677 | 3,7-13 |
| ***RUNX1*** | NM_001754 | ENST00000437180 | all |
| ***SETBP1*** | NM_015559 | ENST00000282030 | 4 |
| ***SF3B1*** | NM_012433 | ENST00000335508 | 10-16 |
| ***SRSF2*** | NM_003016 | ENST00000392485 | 1 |
| ***TET2*** | NM_017628 | ENST00000380013 | all |
| ***TP53*** | NM_000546 | ENST00000269305 | all |
| ***U2AF1*** | NM_006758 | ENST00000291552 | 2,6 |
| ***WT1*** | NM_024426 | ENST00000332351 | 6-10 |
| ***ZRSR2*** | NM_005089 | ENST00000307771 | all |
