# Supplementary material for: Assessment of the clinical utility of four NGS panels in myeloid malignancies. Suggestions for NGS panel choice or design
Source: PLoS One. 2020 Jan 24;15(1):e0227986. doi: 10.1371/journal.pone.0227986 (PMC6980571; doi:10.1371/journal.pone.0227986)
Supplement: S3 Table — SureSeq™ AML panel design includes a total of 20 genes and SureSeq™ CoreMPN panel design includes 3 genes for SNV and indels. (DOCX) [file pone.0227986.s008.docx]

**S3 Table. SureSeq panel target regions per gene.** SureSeq™ AML panel design includes a total of 20 genes and SureSeq™ CoreMPN panel design includes 3 genes for SNV and indels

| **GENE** | **ENSEMBL** | **EXONS Target Region** |
| --- | --- | --- |
| ***ASXL1*** | ENST00000375687 | All |
| ***BCOR*** | ENST00000378444 | All |
| ***CEBPA*** | ENST00000498907 | All |
| ***DNMT3A*** | ENST00000264709 | All |
| ***ETV6*** | ENST00000396373 | All |
| ***FLT3*** | ENST00000241453 | All |
| ***GATA1*** | ENST00000376670 | All |
| ***IDH1*** | ENST00000415913 | All |
| ***IDH2*** | ENST00000330062 | All |
| ***KIT*** | ENST00000288135 | All |
| ***KMT2A/MLL*** | ENST00000534358 | All |
| ***KRAS*** | ENST00000256078 | All |
| ***NPM1*** | ENST00000296930 | All |
| ***NRAS*** | ENST00000369535 | All |
| ***PHF6*** | ENST00000332070 | All |
| ***RUNX1*** | ENST00000300305 | All |
| ***TET2*** | ENST00000540549 | All |
| ***TP53*** | ENST00000269305 | All |
| ***WT1*** | ENST00000332351 | All |
| ***U2AF1*** | ENST00000291552 | All |
| ***MPL*** | ENST00000372470 | 10 |
| ***CALR*** | ENST00000316448 | 9 |
| ***JAK2*** | ENST00000381652 | 12,13,14 |
