# Supplementary material for: Assessment of the clinical utility of four NGS panels in myeloid malignancies. Suggestions for NGS panel choice or design
Source: PLoS One. 2020 Jan 24;15(1):e0227986. doi: 10.1371/journal.pone.0227986 (PMC6980571; doi:10.1371/journal.pone.0227986)
Supplement: S4 Table — PMP panel design includes a total of 48 genes for SNV and indels. (DOCX) [file pone.0227986.s009.docx]

**S4 Table. Pan Myeloid Panel (PMP) target regions per gene.** PMP panel design includes a total of 48 genes for SNV and indels.

| **GENE** | **Transcript** | **ENSEMBL** | **EXONS Target Region** |
| --- | --- | --- | --- |
| ***ANKRD26*** | NM_014915 | ENST00000376087.4 | all |
| ***ASXL1*** | NM_015338.5 | ENST00000375687 | 12 |
| ***ATRX*** | NM_000489.4 | ENST00000373344 | 8-10, 17-31 |
| ***BCOR*** | NM_001123385 | ENST00000378444 | all |
| ***BCORL1*** | NM_021946 | ENST00000540052.1 | all |
| ***CALR*** | NM_004343.3 | ENST00000316448 | 9 |
| ***CBL*** | NM_005188.3 | ENST00000264033 | 8-9 |
| ***CEBPA*** | NM_004364.4 | ENST00000498907 | all |
| ***CSF3R*** | NM_156039 | ENST00000373103 | 14-17 |
| ***CSNK1A1*** | NM_001025105 | ENST00000515768 | all |
| ***CUX1*** | NM_001202543 | ENST00000360264 | all |
| ***DDX41*** | NM_016222 | ENST00000507955.1 | all |
| ***DNMT3A*** | NM_175629.2 | ENST00000264709 | all |
| ***ETNK1*** | NM_018638.4 | ENST00000266517.8 | 3 |
| ***ETV6*** | NM_001987.4 | ENST00000396373 | all |
| ***EZH2*** | NM_004456.4 | ENST00000320356 | all |
| ***FLT3*** | NM_004119.2 | ENST00000241453 | 14-16, 20 |
| ***GATA1*** | NM_002049.3 | ENST00000376670 | 2 |
| ***GATA2*** | NM_032638.4 | ENST00000341105 | 2-6 |
| ***IDH1*** | NM_001282387 | ENST00000415913 | 4 |
| ***IDH2*** | NM_002168.3 | ENST00000330062 | 4 |
| ***IKZF1*** | NM_006060.5 | ENST00000331340 | all |
| ***JAK2*** | NM_004972.3 | ENST00000381652 | 12-15 |
| ***KIT*** | NM_000222.2 | ENST00000288135 | 2,8-11,13,14,17,18 |
| ***KMT2A/MLL*** | NM_001197104.1 | ENST00000534358 | 1-9, 27 |
| ***KRAS*** | NM_033360 | ENST00000256078 | 2-4 |
| ***MPL*** | NM_005373.2 | ENST00000372470 | 3-6, 10, 12 |
| ***NF1*** | NM_001042492 | ENST00000358273 | all |
| ***NPM1*** | NM_002520.6 | ENST00000296930 | 10, 11 |
| ***NRAS*** | NM_002524.4 | ENST00000369535 | 2-4 |
| ***PHF6*** | NM_032458 | ENST00000332070 | all |
| ***PPM1D*** | NM_003620 | ENST00000305921.3 | all |
| ***PTPN11*** | NM_002834.3 | ENST00000351677 | 3, 7, 13 |
| ***RAD21*** | NM_006265.2 | ENST00000297338 | all |
| ***RUNX1*** | NM_001754.4 | ENST00000437180 | all |
| ***SETBP1*** | NM_015559.2 | ENST00000282030 | 4 (aa850-928) |
| ***SF3B1*** | NM_012433.2 | ENST00000335508 | 11-16 |
| ***SH2B3/LNK*** | NM_005475 | ENST00000341259.6 | all |
| ***SMC1A*** | NM_006306 | ENST00000322213 | 2, 11, 16, 17 |
| ***SMC3*** | NM_005445 | ENST00000361804 | 10,13,19,23,25,28 |
| ***SRP72*** | NM_006947 | ENST00000342756.5 | all |
| ***SRSF2*** | NM_003016.4 | ENST00000392485 | 1 |
| ***STAG2*** | NM_001042749.2 | ENST00000218089 | all |
| ***TET2*** | NM_001127208 | ENST00000380013 | all |
| ***TP53*** | NM_000546.5 | ENST00000269305 | all |
| ***U2AF1*** | NM_006758 | ENST00000291552 | 2, 6 |
| ***WT1*** | NM_024426.4 | ENST00000332351 | 7, 9 |
| ***ZRSR2*** | NM_005089.3 | ENST00000307771 | all |
