# Supplementary material for: Assessment of the clinical utility of four NGS panels in myeloid malignancies. Suggestions for NGS panel choice or design
Source: PLoS One. 2020 Jan 24;15(1):e0227986. doi: 10.1371/journal.pone.0227986 (PMC6980571; doi:10.1371/journal.pone.0227986)
Supplement: S5 Table — (DOCX) [file pone.0227986.s010.docx]

| **GENE** | **P** | **M** | **S** | **T** | **MDS/CMML** | **AML/sAML** | **MPN Ph^-^** | **REFERENCE** |
| --- | --- | --- | --- | --- | --- | --- | --- | --- |
| *ABL1* |  | X |  | X |  | CML |  | [1] |
| *ANKRD26* | X |  |  |  | Germline Thrombocytopenia |  |  | [2] [3] [4] [5] |
| *ASXL1* | X | X | X | X | MDS 11-20%/ CMML 47% | AML 10-17%/ sAML 32% | PV 2%/ ET 2-5%/ PMF 10-35%/ aCML 20-30% | [6] [7] [8] [9] [10] [11] [12] [13] |
| *ATRX* | X |  |  | X | 0,8% MDS/ MDS + microcytosis 43% |  |  | [14] |
| *BCOR* | X |  | X | X | MDS 4%/ CMML 7% | AML 7% |  | [15] [16] [17] |
| *BCORL1* | X |  |  | X | MDS <1%/ CMML <1% | AML 2-6% |  | [15] [16] [17] [18] |
| *BRAF* |  | X |  | X |  | AML 1% |  | [19] [1] [20] [21] |
| *CALR* | X | X | X | X | MDS <1%/ CMML <1% |  | PV 0%/ ET 26%/ PMF 20-30% | [22] [10] [21] |
| *CBL* | X | X |  | X | MDS 2-5% / CMML 10-20% /JMML | AML 2%/ sAML 5% | PV 1%/ ET 1%/ PMF 5-10/, aCML 8% | [7] [10] [12] [23] |
| *CBLB* |  |  |  | X |  |  |  | [24] |
| *CBLC* |  |  |  | X |  |  |  | [25] [26] [27] |
| *CDKN2A* |  |  |  | X |  |  |  | [1] [28] |
| *CEBPA* | X | X | X | X | MDS<5%/ CMML 4-20% | AML 6-10%/ biCEBPA AML 1-5% | PV 6%/ ET 4%/ PMF 9%/ aCML 4% | [2] [29] [30] |
| *CSF3R* | X | X |  | X | MDS <1% / CMML 2% | AML 2% | PV 3%/ ET 3%/ PMF 4%/ aCML <10%/ CNL 65% | [31] [12] [21] [32] |
| *CSNK1A1* | X |  |  |  | MDS 5-10%/ CMML <1% |  |  | [33] [34] |
| *CUX1* | X |  |  | X | MDS 2%/ CMML 10% | AML 1% | MPN<3% | [35] [36] |
| *DDX41* | X |  |  |  | Germline | Germline |  | [2] [5] [3] [37] |
| *DNMT3A* | X | X | X | X | MDS 12-18%/ CMML 2-10% | AML 18-36%/ sAML 19% | PV 5-10%/ ET 6%/ PMF 8-12% | [38] [39] [8] [9] [10] [12] [40] [41] [42] [43] |
| *ETNK1* | X |  |  |  | CMML 3% |  | aCML 9% | [44] [45] [46] |
| *ETV6* | X | X | X | X | MDS <5%/ CMML <1% | AML 2% | MPN <3% | [2] [7] [5] [3] [47] |
| *EZH2* | X | X |  | X | MDS 5-10% / CMML 5-12% | AML 4%/ sAML 9% | ET 3%/ PMF 1%/ aCML 13% | [7] [8] [11] [41] |
| *FBXW7* |  |  |  | X |  |  |  | [1] |
| *FLT3* | X | X | X | X | MDS <5%/ CMML <5% | AML 32-39%/ sAML 19% | aCML 1-3%/ PV 1%/ ET 1% | [29] [48] [10] [21] [40] [42] [49] |
| *GATA1* | X |  | X | X |  | Down Syndrome | Down Syndrome | [50] |
| *GATA2* | X |  |  | X | MDS <5%/ CMML <1% | AML 4% | PMF 3% | [2] [51] [5] [52] |
| *GNAS* |  |  |  | X | MDS 2% |  |  | [53] |
| *HRAS* |  | X |  | X |  | Rare |  | [54] |
| *IDH1* | X | X | X | X | MDS <5%/ CMML <1% | AML 7-14% | PMF 1% | [48] [42] [43] [55] [56] |
| *IDH2* | X | X | X | X | MDS<5%/ CMML 5-10% | AML 8-18% | ET 1%/ PMF 3% | [19] [48] [10] [21] [42] [43] |
| *IKZF1* | X |  |  | X |  |  |  | [57] [41] |
| *JAK2* | X | X | X | X | MDS<5%/ CMML 2-10% | AML 1% | PV 98%/ ET 50-60%/ PMF 61% | [9] [10] [21] [58] |
| *JAK3* |  |  |  | X | JMML |  |  | [23] [59] |
| *KDM6A* |  |  |  | X | 1% MDS/ 8% CMML |  |  | [60] |
| *KIT* | X | X | X | X | MDS <3%/ CMML <1% | AML 5% | PV 3%/ ET 2%/ PMF 1% | [61] [19] [10] [21] |
| *KMT2A* | X |  | X | X |  | 5% AML PTD | PV 13%/ PMF 3% | [29] [62] |
| *KRAS* | X | X | X | X | MDS 5-10%/ CMML 10-20% JMML | AML 5%/ sAML 8% | aCML 10% | [29] [38] [7] [62] [10] [12] [13] |
| *MPL* | X | X | X | X | MDS <1%/ CMML <1% |  | ET 3-5%/ PMF 5-10% | [63] [21] |
| *MYD88* |  |  |  | X |  |  |  | [64] [1] [65] |
| *NF1* | X |  |  |  | MDS <9%/ CMML <5%/JMML | AML 3-23%/ sAML 6% |  | [66] [12] [24] [67] |
| *NOTCH1* |  |  |  | X | CMML <1% |  |  | [1] [68] [69] |
| *NPM1* | X | X | X | X | MDS <5%/ CMML <5% | AML 25-35% |  | [29] [38] [8] |
| *NRAS* | X | X | X | X | MDS 5-10%/ CMML 10-20%/ JMML | AML 12-22% | ET 1%,/ PMF 4%/ aCML 10-30% | [38] [7] [12] [13] |
| *PDGFRA* |  |  |  | X |  | AML with eosinophilia |  | [2] [70] [71] |
| *PHF6* | X |  | X | X | MDS 3%/ CMML 4% | AML 3%/ sAML 15% | PV 2% | [39] [72] [73] |
| *PPM1D* | X |  |  |  | MDS 3%, MDS post-HCT 15% |  |  | [74] [58] |
| *PTEN* |  |  |  | X |  |  |  | [1] |
| *PTPN11* | X | X |  | X | MDS <1%/ CMML 4% | AML 10%/ sAML 5%/ tAML 12% | ET 2%/ PMF 2% | [10] [45] [75] |
| *RAD21* | X |  |  | X | MDS 1% | AML 5% |  | [7] [76] [77] |
| *RUNX1* | X | X | X | X | MDS 10-15%/ CMML 10-30% | AML 5-15%/ sAML 31% | PV 2%/ ET 2%/ PMF 4% | [2] [38] [5] [8] [10] [12] [60] |
| *SETBP1* | X | X |  | X | MDS <5-%/ CMML 5-10%/JMML |  | PV 2%/ ET 2%/ PMF 4%/ aCML 25%/ CNL 25% | [78] [7] [10] [23] [60] [79] [80] [81] |
| *SF3B1* | X | X |  | X | MDS 20-30%/ RARS 80% CMML 5-10% | AML 3%/ sAML 11% | PV 3%/ ET 5%/ PMF 5% | [82] [7] [8] [9] [10] [21] |
| *SH2B3* | X |  |  |  | CMML 5% |  | PV 1-2%/ ET 3-6%/ PMF 3-6% | [83] [25] [41] |
| *SMC1A* | X |  |  | X |  |  |  | [76] [77] |
| *SMC3* | X |  |  | X | MDS 1% | AML 3% |  | [7] [76] [77] [60] |
| *SRP72* | X |  |  |  | Germline AA |  |  | [5] [3] |
| *SRSF2* | X | X |  | X | MDS 10-15%/ CMML 30-50% | AML 10%/ sAML 20% | PV 3%/ ET 2%/ PMF 18% | [6] [7] [8] [10] [13] [80] |
| *STAG2* | X |  |  | X | MDS 5-10%/ CMML 5-10% | AML 1%/ sAML 14% |  | [7] [76] [77] [8] [12] [40] |
| *TET2* | X | X | X | X | MDS 20-25%/ CMML 45-60% | AML 14-34%/ sAML 20% | PV 10-20%/ ET 5%/ PMF 10-20% | [6] [29] [8] [9] [10] [13] [41] [80] [84] |
| *TP53* | X | X | X | X | MDS 5-10%/ CMML 10-20% | AML 6-20%/ sAML 15% | PV 1%/ ET 2%/ PMF 1% | [19] [8] [10] [40] [49] [58] [84] |
| *U2AF1* | X | X | X | X | MDS 8-12%/ CMML <5% | AML 4%/ sAML 16% | ET 1%/ PMF 2-8% | [6] [61] [63] [7] [10] [85] |
| *WT1* | X | X | X | X | MDS <3%/ CMML <3% | AML 7-13% |  | [40] [86] |
| *ZRSR2* | X | X |  | X | MDS 5-10%/ CMML 5-10% | AML 2%/ sAML 8% | PV 5%/ ET 3%/ PMF 1% | [61] [38] [7] [10] [81] |
| Del(5q) | X |  |  |  | MDS 3-30% | AML 9%/ sAML 18% | MPN 1-5% | [87] [61] [1] [56] [88] [89] |
| Del(7q)/-7 | X |  |  |  | MDS 1-20%/JMML | AML 1-6% /sAML 8-20% | PMF 7% | [87] [56] [89] [90] |
| Del(20q) | X |  |  |  | MDS 1-7% | AML<1% / sAML 7% | PV<1%/ PMF 24% | [87] [56] [89] [90] [91] |
| Trisomy 8 | X |  |  |  | MDS 1-16% | AML 2-12%/ sAML 10% | PV<1% | [87] [56] [89] [91] |

**S5 Table. Frequency of gene mutations in myeloid malignancies.**

P=PMP; M=MYS; S= SureSeq; T=TSMP; MDS= Myelodisplastic Syndrome; CMML= Chronic Myelomonocytic Leukemia; ; AML= Acute Myeloid Leukemia; sAML= secondary Acute Myeloid Leukemia; MPN=Myeloproliferative Neoplasm; Ph^-^= Philadelphia negative; CML=Chronic Myeloid Leukemia; PV=Polycythemia Vera; ET=Essential Thrombocytopenia; PMF=Primary Myelofibrosis; aCML= atypical Chronic Myeloid Leukaemia; JMML=Juvenile Myelomonocytic Leukemia; tAML=therapy related Acute Myeloid Leukemia; post-HCT= post hematopoietic stem cell transplantation; CNL=Chronic Neutrophilic Leukemia; MDS-RS=Myelodisplastic Syndromes with Ring Sideroblast

**S5 Table References**

1. Taylor J, Xiao W, Abdel-wahab O. Diagnosis and classification of hematologic malignancies on the basis of genetics. Blood. 2017;130: 410–424. doi:10.1182/blood-2017-02-734541.

2. Arber DA, Orazi A, Hasserjian R, Borowitz MJ, Beau MM Le, Bloomfield CD, et al. The 2016 revision to the World Health Organization classi fi cation of myeloid neoplasms and acute leukemia. Blood. 2016;127: 2391–2406. doi:10.1182/blood-2016-03-643544.

3. Babushok D V, Bessler M, Olson TS. Genetic predisposition to myelodysplastic syndrome and acute myeloid leukemia in children and young adults. Leuk Lymphoma. 2016;57: 520–536. doi:10.3109/10428194.2015.1115041

4. Noris P, Perrotta S, Seri M, Pecci A, Gnan C, Loffredo G, et al. Mutationsin ANKRD26 are responsible for a frequent form of inherited thrombocytopenia: analysis of 78 patients from 21 families. Blood. 2011;117: 6673–6680. doi:10.1182/blood-2011-02-336537.

5. Bannon SA, Dinardo CD. Hereditary predispositions to myelodysplastic syndrome. Int J Mol Sci. 2016;17: 838. doi:10.3390/ijms17060838

6. Tefferi A, Lasho TL, Patnaik MM, Saeed L, Mudireddy M, Idossa D, et al. Targeted next-generation sequencing in myelodysplastic syndromes and prognostic interaction between mutations and IPSS-R. Am J Hematol. 2017;92: 1311–1317. doi:10.1002/ajh.24901

7. Pellagatti A, Boultwood J. The molecular pathogenesis of the myelodysplastic syndromes. Eur J Haematol. 2015;95: 3–15. doi:10.1111/ejh.12515

8. Nazha A, Narkhede M, Radivoyevitch T, Seastone DJ, Patel BJ, Gerds AT, et al. Incorporation of molecular data into the Revised International Prognostic Scoring System in treated patients with myelodysplastic syndromes. Leukemia. 2016;30: 2214–2220. doi:10.1038/leu.2016.138

9. Jeromin S, Haferlach T, Weissmann S, Meggendorfer M, Eder C, Nadarajah N, et al. Refractory anemia with ring sideroblasts and marked thrombocytosis cases harbor mutations in SF3B1 or other spliceosome genes accompanied by JAK2V617F and ASXL1 mutations. Haematologica. 2015;100: e125–e127. doi:10.3324/haematol.2014.119032

10. Patnaik MM, Zahid MF, Lasho TL, Finke C, Ketterling RL, Gangat N, et al. Number and type of TET2 mutations in chronic myelomonocytic leukemia and their clinical relevance. Blood Cancer J. 2016;6: e472. doi:10.1038/bcj.2016.82

11. Viny AD, Levine RL. Genetics of myeloproliferative neoplasms. Cancer J. 2014;20: 61–65. doi:10.1097/PPO.0000000000000013

12. Cazzola M, Della Porta MG, Malcovati L. The genetic basis of myelodysplasia and its clinical relevance The genetic basis of myelodysplasia and its clinical relevance. Blood. 2013;122: 4021–4035. doi:10.1182/blood-2013-09-381665

13. McCullough KB, Patnaik MM. Chronic Myelomonocytic Leukemia: a Genetic and Clinical Update. Curr Hematol Malig Rep. 2015;10: 292–302. doi:10.1007/s11899-015-0271-4

14. Herbaux C, Duployez N, Badens C, Poret N, Gardin C, Decamp M, et al. Incidence of ATRX mutations in myelodysplastic syndromes, the value of microcytosis. Am J Hematol. 2015;90: 737–738. doi:10.1002/ajh.24073

15. Terada K, Yamaguchi H, Ueki T, Usuki K, Kobayashi Y, Tajika K, et al. Usefulness of BCOR gene mutation as a prognostic factor in acute myeloid leukemia with intermediate cytogenetic prognosis. Genes Chromosom Cancer. 2018;57: 401–408. doi:10.1002/gcc.22542

16. De Rooij JDE, Van Den Heuvel-Eibrink MM, Hermkens MCH, Verboon LJ, Arentsen-Peters STCJM, Fornerod M, et al. Bcor and bcorl1 mutations in pediatric acute myeloid leukemia. Haematologica. 2015;100: e194–e195. doi:10.3324/haematol.2014.117796

17. Damm F, Chesnais V, Nagata Y, Yoshida K, Scourzic L, Okuno Y, et al. BCOR and BCORL1 mutations in myelodysplastic syndromes and related disorders. Blood. 2013;122: 3169–3177. doi:10.1182/blood-2012-11-469619

18. Rotunno G, Guglielmelli P, Biamonte F, Rumi E, Cazzola M, Vannucchi AM. Mutational analysis of BCORL1 in the leukemic transformation of chronic myeloproliferative neoplasms. Ann Hematol. 2014;93: 523–524. doi:10.1007/s00277-013-1827-9

19. Stenzinger A, Endris V, Pfarr N, Andrulis M, Jöhrens K, Klauschen F, et al. Targeted ultra-deep sequencing reveals recurrent and mutually exclusive mutations of cancer genes in blastic plasmacytoid dendritic cell neoplasm. Oncotarget. 2014;5: 6404–6413. doi:10.18632/oncotarget.2223

20. Xu Y, Wertheim G, Morrissette JJ., Bagg A. BRAF kinase domain mutations in de novo acute myeloid leukemia with monocytic differenciation. Leuk Lymphoma. 2017;58: 743–745. doi:10.1080/10428194.2016.1213830

21. Fei Y, Richard D. Next-Generation Sequencing Multi-Gene Mutation Panels in Myeloid Malignancies. Hematol ASH news reports. 2016;13.

22. Nangalia J, Massie CE, Baxter EJ, Nice FL, Gundem G, Wedge DC, et al. Somatic CALR Mutations in Myeloproliferative Neoplasms with Nonmutated JAK2. N Engl J Med. 2013;369: 2391–2405. doi:10.1056/NEJMoa1312542.

23. Sakaguchi H, Okuno Y, Muramatsu H, Yoshida K, Shiraishi Y, Takahashi M, et al. Exome sequencing identifies secondary mutations of SETBP1 and JAK3 in juvenile myelomonocytic leukemia. Nat Genet. 2013;45: 937–941. doi:10.1038/ng.2698

24. Makishima H, Jankowska AM, Mcdevitt M a, Keefe CO, Dujardin S, Cazzolli H, et al. CBL, CBLB, TET2, ASXL1, and IDH1/2 mutations and additional chromosomal aberrations constitute molecular events in chronic myelogenous leukemia. e-Blood. 2011;117: e198-206. doi:10.1182/blood-2010-06-292433.The

25. Hurtado C, Erquiaga I, Aranaz P, Miguéliz I, García-Delgado M, Novo FJ, et al. LNK can also be mutated outside PH and SH2 domains in myeloproliferative neoplasms with and without V617FJAK2 mutation. Leuk Res. 2011;35: 1537–1539. doi:10.1016/j.leukres.2011.07.009

26. Albitar A, Townsley D, Ma W, De Dios I, Funari V, Young NS, et al. Prevalence of somatic mutations in patients with aplastic anemia using peripheral blood cfDNA as compared with BM. Leukemia. 2018;32: 227–229. doi:10.1038/leu.2017.271

27. Katzav S, Schmitz ML. Mutations of c-Cbl in myeloid malignancies. Oncotarget. 2015;6: 10689–96. doi:10.18632/oncotarget.3986

28. Sulong S, Moorman A V, Irving J a E, Strefford JC, Konn ZJ, Case MC, et al. A comprehensive analysis of the CDKN2A gene in childhood acute lymphoblastic leukemia reveals genomic deletion, copy number neutral loss of heterozygosity , and association with specific cytogenetic subgroups. Blood. 2009;113: 100–107. doi:10.1182/2008-07- 166801

29. Bullinger L, Döhner K, Dohner H. Genomics of acute myeloid leukemia diagnosis and pathways. J Clin Oncol. 2017;35: 934–946. doi:10.1200/JCO.2016.71.2208

30. Fröhling S, Schlenk RF, Stolze I, Bihlmayr J, Benner A, Kreitmeier S, et al. CEBPA mutations in younger adults with acute myeloid leukemia and normal cytogenetics: Prognostic relevance and analysis of cooperating mutations. J Clin Oncol. 2004;22: 624–633. doi:10.1200/JCO.2004.06.060

31. Pardanani A, Lasho TL, Laborde RR, Elliott M, Hanson CA, Knudson RA, et al. CSF3R T618I is a highly prevalent and specific mutation in chronic neutrophilic leukemia. Leukemia. 2013;27: 1870–1873. doi:10.1038/leu.2013.122

32. Kosmider O, Itzykson R, Chesnais V, Lasho T, Laborde R, Knudson R, et al. Mutation of the colony-stimulating factor-3 receptor gene is a rare event with poor prognosis in chronic myelomonocytic leukemia. Leukemia. 2013;27: 1946–1949. doi:10.1038/leu.2013.182

33. Bello E, Pellagatti A, Shaw J, Mecucci C, Kušec R, Killick S, et al. CSNK1A1 mutations and gene expression analysis in myelodysplastic syndromes with del(5q). Br J Haematol. 2015;171: 210–214. doi:10.1111/bjh.13563

34. Meggendorfer M, Haferlach C, Kern W, Haferlach T. Molecular analysis of myelodysplastic syndrome with isolated deletion of the long arm of chromosome 5 reveals a specific spectrum of molecular mutations with prognostic impact: A study on 123 patients and 27 genes. Haematologica. 2017;102: 1502–1510. doi:10.3324/haematol.2017.166173

35. Wong CC, Martincorena I, Rust AG, Rashid M, Alifrangis C, Alexandrov LB, et al. Inactivating CUX1 mutations promote tumorigenesis. Nat Genet. 2014;46: 33–38. doi:10.1038/ng.2846

36. Aly M, Ramdzan’ ZM, Nagata Y, Balasubramanian SK, Hosono N, Makishima H, et al. Functional and Biological Implications of CUX1 Mutations and Deletions in Myeloid Neoplasms. Blood. 2017;130: 120.

37. Lewinsohn M, Brown AL, Weinel LM, Phung C, Rafidi G, Lee MK, et al. Novel germ line DDX41 mutations define families with a lower age of MDS/AML onset and lymphoid malignancies. Blood. 2016;127: 1017–1023. doi:10.1182/blood-2015-10-676098

38. Metzeler KH, Herold T, Rothenberg-Thurley M, Amler S, Sauerland MC, Görlich D, et al. Spectrum and prognostic relevance of driver gene mutations in acute myeloid leukemia. Blood. 2016;128: 686–698. doi:10.1182/blood-2016-01-693879

39. Perry AM, Attar EC. New Insights in AML Biology From Genomic Analysis. Semin Hematol. 2014;51: 282–297. doi:10.1053/j.seminhematol.2014.08.005

40. McCurdi SR, Levis MJ. Emerging molecular predictive and prognostic factors in acute myeloid leukemia. Leuk Lymphoma. 2018;59: 2021–2039. doi:DOI: 10.1080/10428194.2017.1393669

41. Saeidi K. Myeloproliferative neoplasms: Current molecular biology and genetics. Crit Rev Oncol Hematol. 2016;98: 375–389. doi:10.1016/j.critrevonc.2015.11.004

42. Tremblay D, Sokol K, Bhalla S, Rampal R, Mascarenhas JO. Implications of Mutation Profiling in Myeloid Malignancies-PART 1: Myelodysplastic Syndromes and Acute Myeloid Leukemia. Oncol (willinst Park. 2018;32: e38–e44.

43. Im AP, Sehgal AR, Carroll MP, Smith BD, Tefferi A, Johnson DE, et al. DNMT3A and IDH mutations in acute myeloid leukemia and other myeloid malignancies: Associations with prognosis and potential treatment strategies. Leukemia. 2014;28: 1774–1783. doi:10.1038/leu.2014.124

44. Kosmider O. Mutations of ETNK1 in aCML and CMML. Blood. 2015;125: 422–423. doi:10.1182/blood-2014-11-609057

45. Patnaik MM, Barraco D, Lasho TL, Finke CM, Reichard K, Hoversten KP, et al. Targeted next generation sequencing and identification of risk factors in World Health Organization defined atypical chronic myeloid leukemia. Am J Hematol. 2017;92: 542–548. doi:doi:10.1002/ajh.24722

46. Lasho TL, Finke CM, Zblewski D, Patnaik M, Ketterling RP, Chen D, et al. Novel recurrent mutations in ethanolamine kinase 1 (ETNK1) gene in systemic mastocytosis with eosinophilia and chronic myelomonocytic leukemia. Blood Cancer J. 2015;5: e275. doi:10.1038/bcj.2014.94

47. Feurstein S, Godley LA. Germline ETV6 mutations and predisposition to hematological malignancies. Int J Hematol. 2017;106: 189–195. doi:10.1007/s12185-017-2259-4

48. Kantarjian HM, Keating MJ, Freireich EJ. Toward the potential cure of leukemias in the next decade. Cancer. 2018;124: 4301–4313. doi:10.1002/cncr.31669

49. Dohner H, Estey E, Grimwade D, Amadori S, Appelbaum FR, Büchner T, et al. Diagnosis and management of AML in adults: 2017 ELN recommendations from an international expert panel. Blood. 2017;129: 424–447. doi:10.1182/blood-2016-08-733196.424

50. Crispino JD, Horwitz MS. GATA factor mutations in hematologic disease. Blood. 2017;129: 2103–2110. doi:10.1182/blood-2016-09-687889.

51. Hirabayashi S, Wlodarski MW, Kozyra E, Niemeyer CM. Heterogeneity of GATA2-related myeloid neoplasms. Int J Hematol. 2017;106: 175–182. doi:10.1007/s12185-017-2285-2

52. Hahn CN, Chong C-E, Carmichael CL, Wilkins EJ, Brautigan PJ, Li X-C, et al. Heritable GATA2 Mutations Associated with Familial Myelodysplastic Syndrome and Acute Myeloid Leukemia. Nat Genet. 2012;43: 1012–1017. doi:10.1038/ng.913.

53. Di Giacomo D, Lema Fernandez AG, Pierini T, Crescenzi B, Brandimarte L, Matteucci C, et al. The GNAS1 gene in myelodysplastic syndromes (MDS). Leuk Res. 2014;38: 804–807. doi:10.1016/j.leukres.2014.03.017

54. Thomas M, Sukhai MA, Zhang T, Dolatshahi R, Harbi D, Garg S, et al. Integration of Technical, Bioinformatic, and Variant Assessment Approaches in the Validation of a Targeted Next-Generation Sequencing Panel for Myeloid Malignancies. Arch Pathol Lab Med. 2017;141: 759–775. doi:10.5858/arpa.2016-0547-RA

55. Perl AE. The most novel of the novel agents for acute myeloid leukemia. Curr Opin Hematol. 2018;25: 81–89. doi:10.1097/MOH.0000000000000411

56. Milosevic JD, Puda A, Malcovati L, Berg T, Hofbauer M, Stukalov A, et al. Clinical significance of genetic aberrations in secondary acute myeloid leukemia. Am J Hematol. 2012;87: 1010–1016. doi:10.1002/ajh.23309

57. Jäger R, Gisslinger H, Passamonti F, Rumi E, Berg T, Gisslinger B, et al. Deletions of the transcription factor Ikaros in myeloproliferative neoplasms. Leukemia. 2010;24: 1290–1298. doi:10.1038/leu.2010.99

58. Lindsley RC, Saber W, Mar BG, Redd R, Wang T, Haagenson MD, et al. Prognostic Mutations in Myelodysplastic Syndrome after Stem-Cell Transplantation. N Engl J Med. 2017;376: 536–547. doi:10.1056/NEJMoa1611604

59. Assi R, Benton CB, Rawi A Al, Chamoun K, Wang F, Pierce S, et al. JAK3 variants in adults with myeloid malignancies and potential for response to JAK3 inhibition. Blood. 2017;130: 1381.

60. Zhang L, Padron E, Lancet J. The molecular basis and clinical significance of genetic mutations identified in myelodysplastic syndromes. Leuk Res. 2015;39: 6–17. doi:10.1016/j.leukres.2014.10.006

61. Gill H, Leung A, Kwong Y-L. Molecular and Cellular Mechanisms of Myelodysplastic Syndrome: Implications on Targeted Therapy. Int J Mol Sci. 2016;17: 440. doi:10.3390/ijms17040440

62. Song J, Hussaini M, Zhang H, Shao H, Qin D, Zhang X, et al. Comparison of the Mutational Profiles of Primary Myelofibrosis, Polycythemia Vera, and Essential Thrombocytosis. Am J Clin Pathol. 2017;147: 444–452. doi:10.1093/ajcp/aqw222

63. Zoi K, Cross NCP. Genomics of myeloproliferative neoplasms. J Clin Oncol. 2017;35: 947–955. doi:10.1200/JCO.2016.70.7968

64. Lee JH, Jeong H, Choi JW, Oh H, Kim Y-S. Clinicopathologic significance of MYD88 L265P mutation in diffuse large B-cell lymphoma: a meta-analysis. Sci Rep. 2017;7: 1–8. doi:10.1038/s41598-017-01998-5

65. Qin SC, Xia Y, Miao Y, Zhu HY, Wu JZ, Fan L, et al. MYD88 mutations predict unfavorable prognosis in chronic lymphocytic leukemia patients with mutated IGHV gene. Blood Cancer J. 2017;7. doi:10.1038/s41408-017-0014-y

66. Boudry-Labis E, Roche-Lestienne C, Nibourel O, Boissel N, Terre C, Perot C, et al. Neurofibromatosis-1 gene deletions and mutations in de novo adult acute myeloid leukemia. Am J Hematol. 2013;88: 306–311. doi:10.1002/ajh.23403

67. Philpott C, Tovell H, Frayling IM, Cooper DN, Upadhyaya M. The NF1 somatic mutational landscape in sporadic human cancers. Hum Genomics. 2017;11: 13. doi:10.1186/s40246-017-0109-3

68. Francis OL, Chaudhry KK, Lamprecht T, Klco JM. Impact of Notch disruption on myeloid development. Blood Cancer J. 2017;7: e598. doi:10.1038/bcj.2017.73

69. Klinakis A, Lobry C, Abdel-Wahab O, Oh P, Haeno H, Buonamici S, et al. A novel tumour-suppressor function for the Notch pathway in myeloid leukaemia. Nature. 2011;473: 230–233. doi:10.1038/nature09999

70. Naeim F, Rao PN, Song SX, Grody WW. Hematologic Neoplasms Associated with Eosinophilia and PDGFRA, PDGFRB, or FGFR1 Rearrangement. Atlas of Hematopathology. 2013. pp. 213–217.

71. Elling C, Erben P, Walz C, Frickenhaus M, Schemionek M, Stehling M, et al. Novel imatinib-sensitive PDGFRA-activating point mutations in hypereosinophilic syndrome induce growth factor independence and leukemia-like disease. Blood. 2011;117: 2935–2943. doi:10.1182/blood-2010-05-286757

72. Mori T, Nagata Y, Makishima H, Sanada M, Shiozawa Y, Kon A, et al. Somatic PHF6 mutations in 1760 cases with various myeloid neoplasms. Leukemia. 2016;30: 2270–2273. doi:10.1038/leu.2016.212

73. Patel JP, Gönen M, Figueroa ME, Fernandez H, Sun Z, Racevskis J, et al. Prognostic Relevance of Integrated Genetic Profiling in Acute Myeloid Leukemia. N Engl J Med. 2012;366: 1079–1089. doi:10.1056/NEJMoa1112304

74. Wong TN, Miller CA, Jotte MRM, Bagegni N, Baty JD, Schmidt AP, et al. Cellular stressors contribute to the expansion of hematopoietic clones of varying leukemic potential. Nat Commun. 2018;9: 1–10. doi:10.1038/s41467-018-02858-0

75. Ok CY, Patel KP, Garcia-Manero G, Routbort MJ, Fu B, Tang G, et al. Mutational Profiling of Therapy-related Myelodysplastic Syndromes and Acute Myeloid Leukemia by Next Generation Sequencing, a Comparison with de novo Diseases. Leuk Res. 2015;39: 348–354. doi:10.1016/j.leukres.2014.12.006

76. Thol F, Bollin R, Gehlhaar M, Walter C, Dugas M, Suchanek KJ, et al. Mutations in the cohesin complex in acute myeloid leukemia: clinical and prognostic implications. Blood. 2014;123: 914–920. doi:10.1182/blood-2013-07-518746

77. Kon A, Shih LY, Minamino M, Sanada M, Shiraishi Y, Nagata Y, et al. Recurrent mutations in multiple components of the cohesin complex in myeloid neoplasms. Nat Genet. 2013;45: 1232–1237. doi:10.1038/ng.2731

78. Makishima H. Somatic SETBP1 mutations in myeloid neoplasms. Int J Hematol. 2017;105: 732–742. doi:10.1007/s12185-017-2241-1

79. Menezes J, Cigudosa JC. Chronic neutrophilic leukemia: A clinical perspective. Onco Targets Ther. 2015;8: 2383–2390. doi:10.2147/OTT.S49688

80. Cui Y, Tong H, Du X, Li B, Gale PR, Qin T, et al. Impact of TET2, SRSF2, ASXL1 and SETBP1 mutations on survival of patients with chronic myelomonocytic leukemia. Exp Hematol Oncol. 2015;4: 14. doi:10.1186/s40164-015-0009-y

81. Pellagatti A, Roy S, Di Genua C, Burns A, McGraw K, Valletta S, et al. Targeted resequencing analysis of 31 genes commonly mutated in myeloid disorders in serial samples from myelodysplastic syndrome patients showing disease progression. Leukemia. 2016;30: 247–250. doi:10.1038/leu.2015.129

82. Lasho TL, Finke CM, Hanson CA, Jimma T, Knudson RA, Ketterling RP, et al. SF3B1 mutations in primary myelofibrosis: Clinical, histopathology and genetic correlates among 155 patients. Leukemia. 2012;26: 1135–1137. doi:10.1038/leu.2011.320

83. Maslah N, Cassinat B, Verger E, Kiladjian JJ, Velazquez L. The role of LNK/SH2B3 genetic alterations in myeloproliferative neoplasms and other hematological disorders. Leukemia. 2017;31: 1661–1670. doi:10.1038/leu.2017.139

84. Ohgami RS, Ma L, Merker JD, Gotlib JR, Schrijver I, Zehnder JL, et al. Next-generation sequencing of acute myeloid leukemia identifies the significance of TP53, U2AF1, ASXL1, and TET2 mutations. Mod Pathol. 2015;28: 706–714. doi:10.1038/modpathol.2014.160

85. Graubert TA, Shen D, Ding L, Okeyo-Owuor T, Cara L, Shao J, et al. RECURRENT MUTATIONS IN THE U2AF1 SPLICING FACTOR IN MYELODYSPLASTIC SYNDROMES. Nat Genet. 2012;44: 53–57. doi:10.1038/ng.1031

86. Krauth MT, Alpermann T, Bacher U, Eder C, Dicker F, Ulke M, et al. WT1 mutations are secondary events in AML, show varying frequencies and impact on prognosis between genetic subgroups. Leukemia. 2015;29: 660–667. doi:10.1038/leu.2014.243

87. Papaemmanuil E, Gerstung M, Malcovati L, Tauro S, Gundem G, Loo P Van, et al. CME Article Clinical and biological implications of driver mutations in myelodysplastic syndromes. Blood. 2013;122: 3616–3627. doi:10.1182/blood-2013-08-518886.

88. Hosono N, Makishima H, Mahfouz R, Przychodzen B, Yoshida K, Jerez A, et al. Recurrent genetic defects on chromosome 5q in myeloid neoplasms. Oncotarget. 2017;8: 6483–6495. doi:10.1038/leu.2014.25

89. Haase D, Germing U, Schanz J, Pfeilstöcker M, Nösslinger T, Hildebrandt B, et al. New insights into the prognostic impact of the karyotype in MDS and correlation with subtypes: evidence from a core dataset of 2124 patients. Blood. 2007;110: 4385–95. doi:10.1182/blood-2007-03-082404

90. Strasser-Weippl K, Steurer M, Kees M, Augustin F, Tzankov A, Dirnhofer S, et al. Prognostic relevance of cytogenetics determined by flourescent in situ hybridization in patients having myelofibrosis with myeloid metaplasia. Cancer. 2006;107: 2801–2806. doi:10.1002/cncr.22318

91. Gangat N, Strand J, Lasho TL, Finke CM, Knudson RA, Pardanani A, et al. Cytogenetic studies at diagnosis in polycythemia vera: Clinical and JAK2V617F allele burden correlates. Eur J Haematol. 2008;80: 197–200. doi:10.1111/j.1600-0609.2007.01003.x
