# Supplementary material for: Assessment of the clinical utility of four NGS panels in myeloid malignancies. Suggestions for NGS panel choice or design
Source: PLoS One. 2020 Jan 24;15(1):e0227986. doi: 10.1371/journal.pone.0227986 (PMC6980571; doi:10.1371/journal.pone.0227986)
Supplement: S6 Table — (DOCX) [file pone.0227986.s011.docx]

**S6 Table. Common sequencing errors detected in the NGS gene panels.**

| **Gene** | **Chr** | **Position** | **CodCons** | **Transcript** | **c.DNA** | **Protein** | **PMP number of samples (%)** | **MYS number of samples (%)** | **SureSeq number of samples (%)** | **TSMP number of samples (%)** |
| --- | --- | --- | --- | --- | --- | --- | --- | --- | --- | --- |
| *ANKRD26* | 10 | 27337805 | inframe_3 | NM_001256053 | c.1736_1738delATG | p.Asp579del | 20 (63%) | NI | NI | NI |
| *ASXL1* | 20 | 31022441 | frameshift | NM_015338.5 | c.1926_1927insG | p.Gly646TrpfsTer12 | 0 (0%) | 0 (0%) | 0 (0%) | 17 (100%) |
| *ASXL1* | 20 | 31022441 | frameshift | NM_015338.5 | c.1927delG | p.Gly645ValfsTer58 | 0 (0%) | 0 (0%) | 16 (100%) | 17 (100%) |
| *ATRX* | X | 76920172 | frameshift | NM_000489.3 | c.3904delA | p.Arg1302GlufsTer44 | 0 (0%) | NI | NI | 17 (100%) |
| *BCORL1* | X | 129190010 | frameshift | NM_021946.4 | c.5035_5036insC | p.Gly1682ArgfsTer4 | 0 (0%) | NI | NI | 12 (71%) |
| *BCORL1* | X | 129190010 | frameshift | NM_021946.4 | c.5036delC | p.Pro1681GlnfsTer20 | 0 (0%) | NI | NI | 12 (71%) |
| *CBL* | 11 | 119149355 | inframe_3 | NM_005188 | c.1380_1382delTGA | p.Asp460del | 32 (100%) | NI | NI | 17 (100%) |
| *CEBPA* | 19 | 33792754 | inframe_3 | NM_004364 | c.564_566delGCC | p.Pro189del | 32 (100%) | 15 (100%) | 14 (88%) | 12 (71%) |
| *CEBPA* | 19 | 33793007 | inframe_3 | NM_004364 | c.311_313delGCG | p.Gly104del | 7 (22%) | 13 (87%) | 8 (50%) | 0 (0%) |
| *CUX1* | 7 | 101892132 | inframe_3 | NM_001202543 | c.4375_4377delAGC | p.Ser1459del | 32 (100%) | NI | NI | 0 (0%) |
| *CUX1* | 8 | 101839973 | frameshift | NM_001202543 | c.1316delC | p.Pro441LeufsTer27 | 0 (0%) | NI | NI | 8 (47%) |
| *IDH2* | 15 | 90631917 | frameshift | NM_002168.2 | c.435dupG | p.Thr146AspfsTer126 | 0 (0%) | 0 (0%) | 0 (0%) | 17 (100%) |
| *IDH2* | 15 | 90631917 | frameshift | NM_002168.2 | c.435delG | p.Thr146LeufsTer15 | 0 (0%) | 0 (0%) | 0 (0%) | 17 (100%) |
| *KMT2A* | 11 | 118307413 | inframe_3 | NM_001197104 | c.200_202delCGG | p.Ala67del | 32 (100%) | NI | NI | 0 (0%) |
| *NOTCH1* | 9 | 139390944 | inframe_3 | NM_017617.3 | c.7244_7246delCAC | p.Pro2415del | NI | NI | NI | 17 (100%) |
| *NOTCH1* | 9 | 139399408 | inframe_3 | NM_017617.3 | c.4732_4734delGTG | p.Val1578del | NI | NI | NI | 12 (71%) |
| *SF3B1* | 2 | 198266476 | frameshift | NM_012433.2 | c.2359delA | p.Ile787LeufsTer3 | 0 (0%) | NI | NI | 5 (30%) |
| *STAG2* | X | 123200048 | missense | NM_001042749 | c.2120A>T | p.Asp707Val | 0 (0%) | NI | NI | 13 (76%) |
| *STAG2* | X | 123200056 | missense | NM_001042749 | c.2128G>T | p.Ala710Ser | 0 (0%) | NI | NI | 9 (53%) |

Chr= chromosome; CodCons= coding consequence; NI= Gene not included in panel design; SNP= Single nucleotide polymorphism
