# Supplementary material for: Assessment of the clinical utility of four NGS panels in myeloid malignancies. Suggestions for NGS panel choice or design
Source: PLoS One. 2020 Jan 24;15(1):e0227986. doi: 10.1371/journal.pone.0227986 (PMC6980571; doi:10.1371/journal.pone.0227986)
Supplement: Attachment — Submitted filename: Response to Reviewers.docx [file pone.0227986.s012.docx]

RE: Manuscript ID PONE-D-19-20399 entitled “Assessment of the clinical utility of four NGS panels in myeloid malignancies. Suggestions for NGS panel choice or design”

**Editor Comments**

**Thank you for including your ethics statement:** **Please amend your current ethics statement to include the full name of the ethics committee/institutional review board(s) that approved your specific study.**

Thank you for pointing out the need of amending the ethics statement. Now it reads as follows: “Samples and data from patients included in the study were provided by the Biobank of the University of Navarra (UN) and were processed following standard operating procedures approved by the CEI (Comité de Ética de la Investigación) of UN. Patient’s data were fully anonymized, and all patients provided informed written consent to have data from their medical records such as age, gender and diagnosis to be used for research purpose.”

**Once you have amended this/these statement(s) in the Methods section of the manuscript, please add the same text to the “Ethics Statement” field of the submission form (via “Edit Submission”).**

The Ethics Statement has also been updated in the submission form

**The Authors have put together a timely study. Unfortunately this manuscripts suffers from a lack of clarity in terms of design and write-up including presentation of the results. This paper would require major revision in order to be considered for publication.**

**Major points:**

**1. Why were a select set of samples not tested across all panels? There needs to be an exact comparison to provide credence to their hypothesis.**

We agree with the Editor that all samples should have been tested across all panels. Unfortunately, we could not run the 32 samples with the four NGS gene panels, due to limited sample availability. We have included in the study only the 32 cases with enough DNA to build libraries with at least two different panels.

**2. What was the reason for extra samples (total of 32) only being tested on the in-house panels.**

Our study includes 3 commercially available panels and only 1 custom (in-house) panel. We have included in the study 32 cases analyzed with at least two different panels. Figure 1 shows which panels (2, 3 or 4) have been used to sequence each sample.

**3. The results should be clearly iterated as well as all points specifically addressed in the discussion.**

We thank the Editor for this suggestion. We think that the manuscript now reads more clear and it is easier to follow the conclusions.

**Minor Points:**

**1. The manuscript needs to be edited for typos, grammar and language.**

We have now carefully reviewed the full manuscript and all typos and grammar issues have been addressed.

**Reviewer #1**

**In this manuscript, the authors investigated four NGS panels in myeloid malignancies. They evaluated three commercially available targeted NGS gene panels and one custom panel based on the technical features and variant detection of myeloid neoplasms related genes. The manuscript is well written. Below are the comments I have:**

**1. The authors collected total 32 patient samples: 17 with AML, 7 with MPN, 6 with MDS, and 2 with CMML in this study. All 32 samples were tested with the custom panel. However, only some of the samples were used for the other three commercial NGS panels. Was there any reason that the authors did not include all 32 samples for testing all four panels? It would be clearer if the authors could provide information on how the samples were selected for each panel testing and what sample types were included.**

The reason why it was not possible to test the 32 samples with all four panels, was material constrains. We have included in the study those cases with enough DNA to build libraries with at least two different panels. When in doubt, what panel to use in a particular sample, we prioritized library construction with the panel which covered previously known variants in a particular sample.

**2. In Table 2, the authors listed the software used for different panel analysis. However, the analysis tool for the custom panel was developed in-house. At the time of this study, was this tool validated for variant calling?**.

Raw data from the custom panel were uploaded onto SOPHiA DDM software, where alignment, variant calling and annotation were performed. SOPHiA Genetics develops custom panels including a panel validation program: firstly, the company performs the panel technical validation in their facilities; secondly, clinical validation is performed in the hospital facilities, including samples with multiple known mutations, aiming at confirming the capability of the SOPHiA DDM software of calling those variants.

**3. The clinical relevance of each gene included in all four panels has been represented in Figure 2. However, I found it difficult to understand the label of Figure 2. It is unclear what the clinical relevancies are for. Is it based on the summary of different clinical relevance categories results?**

We agree with the Reviewer that Figure 2 legend was confusing. We have now rewritten the Figure legend. It explains that clinical relevance of the genes refers to their diagnostic, prognostic and/or predictive value, or their relation to predisposition to develop MN. The legend now reads as follows:

**“Figure 2.** Genes covered by each panel and their clinical relevance. The 62 genes included in the present study are listed on the right. Black color denotes which gene is covered in each panel. Green color highlights the 53 genes that have been described as clinically relevant for MN, since they show diagnostic, prognostic and/or predictive value, or they have been related to predisposition to develop MN. Red color represents genes that are not clinically relevant in MN. Grey color marks those genes that has been described in MN but their clinical relevance is still unknown”

**4. The authors discussed that the sequencing errors of TSMP were likely due to its amplicon-based nature. However, with only one amplicon-based targeted panel included in the comparison, this conclusion is not convincing. In addition, the analysis software of TSMP was different from other panels. It is possible that the variants calling is affected by the software.**

We agree with the Reviewer that only one amplicon-based targeted panel is not enough to conclude anything about amplicon based-panels. Hence, we have now deleted the sentence in which we speculated about the reason of abundant sequencing errors in this panel.

**5. The authors listed 19 genes as the core myeloid gene set. Four references papers were cited in the discussion to support this core gene set. However, it is unclear that how these 19 gene were selected since there were other genes listed in all four papers.**

We thank the Reviewer for raising this point. We have named the 19 genes which are included in all four panels “core myeloid gene set” for the sake of clarity in the analysis. It is only a descriptive term; we do not mean that those are the only genes to be sequenced in MN. In fact, based on an extended literature research that we carried out, we mention in the manuscript the importance of updating the list of genes with clinical relevance in MN.
