# Supplementary material for: Assessment of the clinical utility of four NGS panels in myeloid malignancies. Suggestions for NGS panel choice or design
Source: PLoS One. 2020 Jan 24;15(1):e0227986. doi: 10.1371/journal.pone.0227986 (PMC6980571; doi:10.1371/journal.pone.0227986)
Supplement: Attachment — Submitted filename: Response to Reviewers 2.0.docx [file pone.0227986.s013.docx]

RE: Manuscript ID PONE-D-19-20399 entitled “Assessment of the clinical utility of four NGS panels in myeloid malignancies. Suggestions for NGS panel choice or design”

**Editor Comments**

**We thank the authors for responding to our comments and making the necessary edits. While the technical content of the manuscript is now sound, there still needs to be some clarity and flow to the manuscript to ensure the message is clear. For example in line 41, you refer to NGS as a fundamental instrument, which is not accurate. NGS is a technology. It would help get the message across if the flow of the manuscript is a bit smoother.**

We thank the Editor for raising this point, which has now been taken under consideration. We have adapted the manuscript according to the recommendations, and we believe that the paper now flows smoother.

**Reviewer#1**

There are no additional points raised by Reviewer 1
